# Home Use of a Percutaneous Wireless Intracortical Brain-Computer Interface by Individuals With Tetraplegia

**DOI:** 10.1109/TBME.2021.3069119

**Published:** 2021-06-17

**Authors:** John D. Simeral, Thomas Hosman, Jad Saab, Sharlene N. Flesher, Marco Vilela, Brian Franco, Jessica Kelemen, David M. Brandman, John G. Ciancibello, Paymon G. Rezaii, Emad N. Eskandar, David M. Rosler, Krishna V. Shenoy, Jaimie M. Henderson, Arto V. Nurmikko, Leigh R. Hochberg

**Affiliations:** Center for Neurorestoration and Neurotechnology, Rehabilitation R&D Service, Department of Veterans Affairs Medical Center, Providence, RI 02908 USA (CfNN) and with the School of Engineering and the Robert J. & Nancy D. Carney Institute for Brain Science, Brown University, Providence, RI; CfNN and the School of Engineering, Brown University; CfNN and the School of Engineering, Brown University. He is now with Insight Data Science, New York City, NY; Department of Electrical Engineering, Department of Neurosurgery, and Howard Hughes Medical Institute, Stanford University. She is now with Apple Inc., Cupertino, CA; School of Engineering, Brown University. He is now with Takeda, Cambridge, MA; Department of Neurology, Massachusetts General Hospital, Boston, MA. He is now with NeuroPace Inc., Mountain View, CA; Department of Neurology, Massachusetts General Hospital, Boston; School of Engineering, Brown University. He is now with the Department of Neurosurgery, Emory University, Atlanta, GA; School of Engineering, Brown University. He is now with the Center for Bioelectronic Medicine at the Feinstein Institute for Medical Research, Manhasset, NY; Department of Neurosurgery, Stanford University. He is now with the School of Medicine, Tulane University; Department of Neurosurgery, Massachusetts General Hospital. He is now with the Department of Neurosurgery, Albert Einstein College of Medicine, Montefiore Medical Center, NY; CfNN and the School of Engineering and the Robert J. & Nancy D. Carney Institute for Brain Science, Brown University, and also with the Department of Neurology, Massachusetts General Hospital; Departments of Electrical Engineering, Bioengineering and Neurobiology, Wu Tsai Neurosciences Institute, and the Bio-X Institute, Stanford, and also with the Howard Hughes Medical Institute, Stanford University; Department of Neurosurgery and Wu Tsai Neurosciences Institute and the Bio-X Institute, Stanford University; School of Engineering, Department of Physics, and the Robert J. & Nancy D. Carney Institute for Brain Science, Brown University; CfNN, and the School of Engineering and the Robert J. & Nancy D. Carney Institute for Brain Science, Brown University, and the Center for Neurotechnology and Neurorecovery, Department of Neurology, Massachusetts General Hospital, Harvard Medical School

**Keywords:** Brain-computer interface, clinical trial, motor cortex, neural engineering, wireless transmitter

## Abstract

**Objective.:**

Individuals with neurological disease or injury such as amyotrophic lateral sclerosis, spinal cord injury or stroke may become tetraplegic, unable to speak or even locked-in. For people with these conditions, current assistive technologies are often ineffective. Brain-computer interfaces are being developed to enhance independence and restore communication in the absence of physical movement. Over the past decade, individuals with tetraplegia have achieved rapid on-screen typing and point-and-click control of tablet apps using intracortical brain-computer interfaces (iBCIs) that decode intended arm and hand movements from neural signals recorded by implanted microelectrode arrays. However, cables used to convey neural signals from the brain tether participants to amplifiers and decoding computers and require expert oversight, severely limiting when and where iBCIs could be available for use. Here, we demonstrate the first human use of a wireless broadband iBCI.

**Methods.:**

Based on a prototype system previously used in pre-clinical research, we replaced the external cables of a 192-electrode iBCI with wireless transmitters and achieved high-resolution recording and decoding of broadband field potentials and spiking activity from people with paralysis. Two participants in an ongoing pilot clinical trial completed on-screen item selection tasks to assess iBCI-enabled cursor control.

**Results:**

Communication bitrates were equivalent between cabled and wireless configurations. Participants also used the wireless iBCI to control a standard commercial tablet computer to browse the web and use several mobile applications. Within-day comparison of cabled and wireless interfaces evaluated bit error rate, packet loss, and the recovery of spike rates and spike waveforms from the recorded neural signals. In a representative use case, the wireless system recorded intracortical signals from two arrays in one participant continuously through a 24-hour period at home.

**Significance.:**

Wireless multi-electrode recording of broadband neural signals over extended periods introduces a valuable tool for human neuroscience research and is an important step toward practical deployment of iBCI technology for independent use by individuals with paralysis. On-demand access to high-performance iBCI technology in the home promises to enhance independence and restore communication and mobility for individuals with severe motor impairment.

## I. INTRODUCTION

NEUROLOGICAL disease or injury such as amyotrophic lateral sclerosis, stroke and cervical spinal cord injury (SCI) can result in tetraplegia, loss of speech or locked-in syndrome. Brain-computer interfaces (BCIs) are being developed to restore communication and motor function for individuals living with profound motor disability. Motor BCIs aim to provide access to assistive devices by decoding user commands from a variety of sources including electroencephalography (EEG), electrocorticography (ECoG), or intracortical signals. In ongoing clinical research, high-performance intracortical BCIs (iBCIs) are being developed to infer a user’s movement intentions [1–8] or speech [9], [10] from neural activity recorded from one or more microelectrode arrays implanted in motor areas of cortex. By imagining natural hand, finger and arm movements, trial participants with paralysis have achieved reach-and grasp with robotic and prosthetic limbs [2], [3], [11] and their own reanimated limb [6], [12], and have demonstrated reliable cursor control for tablet use [8] and on-screen typing for communication [4], [7], [13]. Building on steady advances in point-and-click accuracy, speed [4], [7], [13–16] and consistency [4], [17–20], iBCI trial participants at home have achieved average on-screen point-to-select typing rates over 37 correct characters per minute maintained over days and weeks [4], [7].

While this progress is promising, one practical limitation of current iBCIs is their reliance on recording cables that link an implanted array’s head-mounted titanium connector (“pedestal”) to the signal processing and decoding computers. Enabling iBCIs for long-term recording and independent mobile use at home without technical supervision will require wireless acquisition of intracortical signals to eliminate tethering cables to the head. However, wireless recording for iBCI has yet to be demonstrated in people, in part because translating the proven cabled system (16 bits per sample at 30 kS/s for each of 96 electrodes per implanted array) to wireless form presents significant engineering challenges. Although wireless broadband signal acquisition might not be a design requirement for some targeted iBCI applications [21–23], broadcasting the entire broadband signal allows for investigation of novel iBCI algorithms spanning the full spectrum of neural activity while supporting a wide range of fundamental electrophysiological research during untethered use. With this motivation and the goal of enabling continuous, independent use of an iBCI, previous work from our team created a compact, power efficient neurosensor that digitizes and wirelessly transmits broadband cortical activity from a 96-channel chronically implanted microelectrode array [24–27]. A battery-powered pedestal-mounted form of that transmitter was demonstrated in animals during open-loop tasks and free home-cage behavior [24], [25]. A fully implanted inductively-charged version was developed and tested *in-vivo* in non-human primates [26], [27] and is on a translational path for human use. Both the external and fully implanted devices were designed with a relatively high sample rate (20 kS/s per electrode, 12 bits/sample) to support both fundamental human neuroscience research and investigational signal processing and decoding methods for high-performance iBCI systems.

Here we report translation of a wireless broadband intracortical BCI system to human use and evaluate its performance during use at home by two participants in the BrainGate pilot clinical trial. The external transmitter underwent commercial manufacturing and preclinical safety testing in preparation for human investigational use. Transmission frequency was configurable such that neural activity from two 96-channel intracortical arrays could be recorded simultaneously without interference. Two transmitters and associated commercial receiver hardware were integrated into the iBCI real-time signal processing system. Both participants then used the BrainGate system to complete a series of cursor-based point-and-dwell assessment tasks [1], [7], [28] to quantify iBCI performance in cabled and wireless configurations. We demonstrate that the wireless signals could be decoded with sufficient quality and reliability to support spontaneous user-paced point-and-click use of a tablet computer as reported for previous participants using a cable connection [8]. To verify wireless function in one representative iBCI use scenario, one participant completed dual-array wireless recording over 24 hours of daily activity, rest and sleep. Finally, we examined the underlying signal quality and digital sample integrity in the wireless data streams relative to the cabled system in both benchtop and in-home evaluations.

## II. Methods

### A. Participants

Study participants T5 and T10 were previously enrolled in the pilot clinical trial of the BrainGate Neural Interface System. Enrollment criteria and other details about the trial are available at (http://www.clinicaltrials.gov/ct2/show/NCT00912041). At the time of this study, T5 was a 63-year-old man with a C4 AIS-C spinal cord injury resulting in tetraplegia. T10 was a 35-year-old man with a C4 AIS-A SCI resulting in tetraplegia. Prior to this study, each participant had engaged in approximately 100 research sessions that spanned a wide variety of study designs including many that focused on the development of novel neural decoders and iBCI cursor control using some of the tasks employed in this study. The participants had little or no prior experience using the wireless system (2 sessions for T10, none for T5).

All activities, including use of the wireless devices, were permitted by the US Food and Drug Administration (FDA, Investigational Device Exemption #G090003) and the Institutional Review Boards (IRBs) of Partners Healthcare/Massachusetts General Hospital (#2009P000505), Providence VA Medical Center, Stanford University and Brown University.

For each participant, intracortical neural activity was recorded from two 96-channel planar silicon microelectrode arrays (4 mm x 4 mm, 1.5 mm electrode length, platinum tips; Blackrock Microsystems, Salt Lake City, UT) placed in the left (dominant) precentral gyrus, except participant T10 whose second array was placed in the middle frontal gyrus.

### B. Standard Cabled iBCI System

**Fig. 1.**
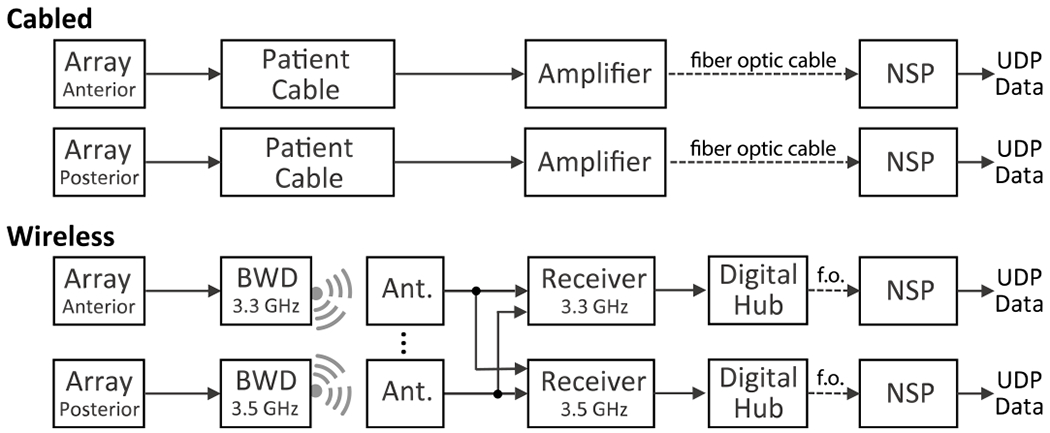
Components of the cabled and wireless systems for dual-array recording. Pathways for neural signal acquisition differed as shown, but NSPs and all downstream file recording, signal processing and decoding hardware and software were the same for both systems. Ant.: antenna; f.o.: fiber optic.

The cabled BrainGate iBCI used commercial hardware and software (Blackrock Microsystems) to acquire and record neural signals. This system included a NeuroPort Patient Cable connecting each percutaneous head-mounted pedestal to a Front End Amplifier which applied a hardware filter (0.3 Hz – 7.5 kHz) and digitized signals on each of 96 microelectrodes (30 kS/s, 16 bits per sample). The continuous serial stream of digital samples was relayed over fiber optic cable to a Neural Signal Processor (NSP) where they were timestamped and sent out as UDP packets on a private local area network. These “raw” data were stored (without software filtering or down-sampling) by Blackrock’s Central Suite software for offline analysis. Because participants in this study each had two arrays, the home iBCI included two parallel 96-channel acquisition systems (Fig. 1, top) that were time-locked by a sync cable linking the two NSPs. Raw data packets were delivered to the remainder of the investigational BrainGate system for wireless control of communication devices (see Supplemental Methods).

### C. Pedestal-Mounted Wireless Transmitter (BWD)

**Fig. 2.**
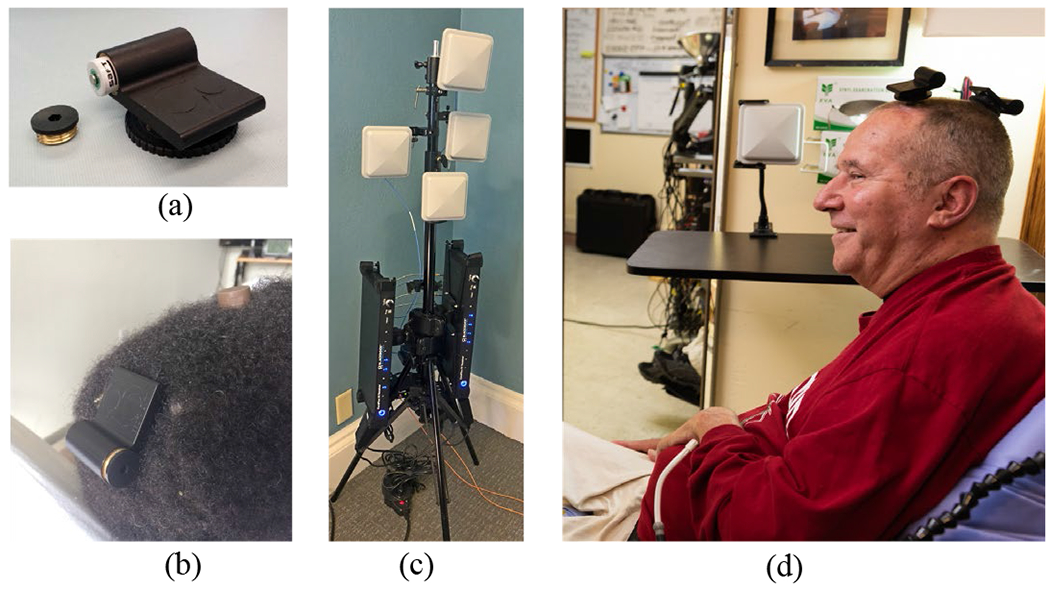
Some components of the wireless system, (**a**) BWD transmitter (52 mm x 44 mm) showing battery compartment. Turn-screw disc is used to attach the device onto a percutaneous pedestal, (**b**) The BWD connected to T10’s posterior pedestal (here, the anterior pedestal is covered by a protective cap), (**c**) A two-frequency wireless receiver system in a four-antenna configuration as deployed for T10. The output optical fibers (orange) connect to downstream NSPs. (**d**) T5 in his home with two transmitters. The antenna in the background was one of four mounted around the room. Photos used with permission.

The neurosensor deployed here (Fig. 2a, b) was designed from the outset for translation to human intracortical recording and in-home iBCI applications. Design criteria included a high digital sampling rate to capture neuronal spiking activity, data acquisition and transmission over at least 24 hours for use throughout the day, and lightweight mating to the percutaneous pedestal that is currently the only interface available for chronic intracortical microelectrode recording in people (research only). It was designed as a low-power, short-range (meters) broadband device operating in a low-use portion of the microwave communications spectrum. Details of the device design and characterization have been reported previously for external [24], [25] and fully implanted [26], [27] forms. The external device was licensed to Blackrock Microsystems who made minor design refinements and manufactured it as the “Brown Wireless Device” (BWD). The BWD applies a hardware filter (1 Hz to 7.8 kHz), digitizes neural activity (20 kS/sec, 12 bits per sample) on each electrode, applies Manchester encoding and transmits it to nearby antennas using a custom low-power protocol. The Manchester encoding provides self-clocking, reduces link error rate and enhances reliability. In the digital data stream, a 24-bit “sync word” separates each frame of data containing one 50 μs 12-bit sample from all electrodes. BWDs use a non-rechargeable medical-grade battery (SAFT LS14250). Key BWD device specifications are summarized in Supplemental Table SI. Pre-clinical device safety assessments of the BWD were completed prior to use in the BrainGate trial.

### D. Wireless iBCI System

In the wireless recording system (Fig. 1, bottom), each NeuroPort Patient Cable and Front End Amplifier was replaced by four components (Blackrock Microsystems): a BWD, one or more polarized planar antennas (5” x 5”, 3 GHz – 4 GHz reception, PA-333810-NF), a Wireless Receiver (PN9323) and a Digital Hub (PN6973). Each BWD digitized neural activity from one array and transmitted it at 3.3 GHz or 3.5 GHz (configured at time of manufacture) to the antennas. The corresponding Wireless Receiver was manually tuned to the appropriate frequency and could detect valid data packets (frames) received by any of up to 8 input antennas (this study used 4 antennas shared between the receivers, except 8 in the 24-hour study). Each Digital Hub relayed the digital data stream to its respective NSP over fiber optic cable. The NSP, file recording system, and downstream hardware and software were unchanged between the wired and wireless configurations.

As with the wired system, dual-array wireless recording required two parallel sets of equipment, except that the SMA cable from each antenna was split and connected to both wireless receivers. This avoided the need for duplicate antennas at the cost of splitting the signal power between the receivers.

To establish compatibility with the downstream data recording system used for this study, the wireless hardware (Wireless Receiver / Digital Hub) upsampled the BWD data stream from 20 kS/s to 30kS/s (via sample-and-hold) and from 12 bits per sample to 16 bits per sample (four-bit up-shift). The upsampled wireless data was then stored in the standard ns5 file format and processed through the identical signal processing software as the cabled data. We applied a variety of analyses to assess the effect of these sample-level transformations on recorded neural signal content and iBCI decoder function.

Additional details of the wireless methods are provided in the Supplementary Materials.

### E. Decoded Cursor Control in Cabled versus Wireless iBCI

Research sessions took place in participants’ homes with oversight by each site’s trained clinical neurotechnology research assistant (CNRA). T5 completed study activities seated in a wheelchair in his living room. For health reasons unrelated to the neural interface system, T10 completed all sessions while reclined in bed. Sessions lasted approximately 3 hours, interrupted as needed by the participant’s regular nursing and personal care activities.

Participant sessions in this study included T10 trial days (post-implant day) 307, 349, 350, 355, 361 and T5 trial days 560, 572, 588. Different study tasks were completed on different days. Each participant completed two sessions (T10 trial days 355, 361; T5 days 560, 572) in which cabled versus wireless cursor performance was assessed in a series of interleaved back-to-back point-and-select Grid Tasks described previously [1], [7], [29]. Performance was assessed for each 2-minute task in terms of Percent Correct target acquisitions, achieved Bitrate, Trial Duration, Path Efficiency and Angle Error. These sessions were also used to evaluate recorded signal quality, packet loss, and other measures comparing the wired and wireless conditions (see below).

On different days, both participants also used the wireless system to navigate consumer software and web applications on a tablet computer (T10 day 307; T5 day 588), during which T5 also performed typing tasks.

For iBCI cursor tasks, a Kalman filter decoder and a linear discriminant classifier were used to estimate continuous 2-D cursor velocity and click state, respectively. After upsampling in the wireless path (see above), signal processing and decoding algorithms (described in detail previously [4]) were identical for cabled and wireless conditions.

### F. 24-Hour Wireless Recording

T10 also completed an overnight in-home study to collect nearly continuous intracortical data over a 24-hour period (days 349, 350). During this time, T10 remained in bed for reasons independent of this study, but he engaged in his typical daily activities such as eating, watching television, conversing by phone, direct family interactions, sleeping, etc. He received his regular nursing care including rotations in bed every few hours. The quality and data integrity of the recorded data was analyzed in 5-minute segments contiguously across the 24-hour period.

### G. Comparing Cabled and Wireless Signal Fidelity

We conducted a series of A/B experiments to more directly compare the fidelity of broadband recordings using the standard 96-channel NeuroPort Patient Cable “reference system” and the 96-channel wireless system. These tests were performed with simulated neural signals in the lab using a Neural Signal Simulator (Blackrock Microsystems) and with intracortical signals recorded in the home. We compared noise, bandpass filtered signals, sorted spike waveforms and unit firing rates.

Transmission data loss is a concern for all wireless systems. Here, wireless data (frame) loss occurred whenever the receiver did not find the expected digital sync word on the incoming wireless data stream from at least one antenna. When this occurred, the current samples from all 96 electrodes were invalid and the previous valid frame was inserted into the data stream. We quantified the incidence and impact of these frame errors during iBCI decoding and during the 24-hour study.

Additional details describing the neural decoding methods, assessments Grid Task, wireless iBCI use of a tablet computer, 24-hour wireless recording, and digital sample integrity measures appear in the Supplementary Materials.

## III. Results

### A. Translation of the Wireless Sensor for Human Studies

The commercialized BWD underwent pre-clinical device testing in accordance with FDA Good Laboratory Practice (GLP) guidelines. Testing to IEC / ISO standards was completed under the direction of Blackrock Microsystems which coordinated with accredited test service providers. Testing verified BWD immunity to repeated direct and indirect electrostatic discharge (ESD) up to +/−6kV and +/−8kV, respectively (per IEC 61000-4-2). Testing also found that charge transients, charge per phase, and charge density at the electrodes remained well within safety thresholds during BWD power transitions and uneven power connection. Together, these tests confirmed that the BWD effectively limited charge transients through the recording electrodes under various conditions. Considerations for electrical safety design of the wireless transmitter are discussed further in Yin et al. [25].

For clinical applications, ethylene oxide (EtO) is a low-temperature gaseous sterilization process appropriate for sensitive electronics. BWD testing verified that EtO sterilization was complete (per ANSI/AAM/ISO 10993-7-2008) and did not compromise the BWD electronics. BWDs are sterilized and stored in sterile packaging prior to use.

The BWD uses a very low power transmitter. Radio frequency (RF) testing verified safety of the BWD transmission which measured well under 1 mW, well below the specific absorption rate (SAR) exclusion threshold of 16 mW power specified in the FCC General RF Exposure Guidance (FCC 447498 D01). In further testing, the transmitter was operated in high density RF environments ranging from a major hospital to a metropolitan city center with no interference from such telecommunications-rich environments. The FCC granted an Experimental License to operate the BWD in the 3.3 GHz – 3.8 GHz transmission band in geographical regions related to the BrainGate trial.

### B. Assessment of Wireless Point and Click Cursor Control

We evaluated if the cabled and wireless iBCI configurations enabled equivalent kinematic decoding and closed-loop cursor performance by BrainGate trial participants in their homes. On each of two days, participants successfully achieved cursor control using an automated calibration procedure which generated the Kalman decoder coefficients used in the subsequent 6×6 Grid Task assessment blocks. Across two sessions, T10 completed 251 trials over 6 blocks in the cabled configuration and 235 trials over 6 blocks in the wireless configuration. Participant T5 completed two sessions totaling 912 trials over 20 blocks in the cabled configuration and 1094 trials over 24 blocks in the wireless configuration.

**Fig. 3.**
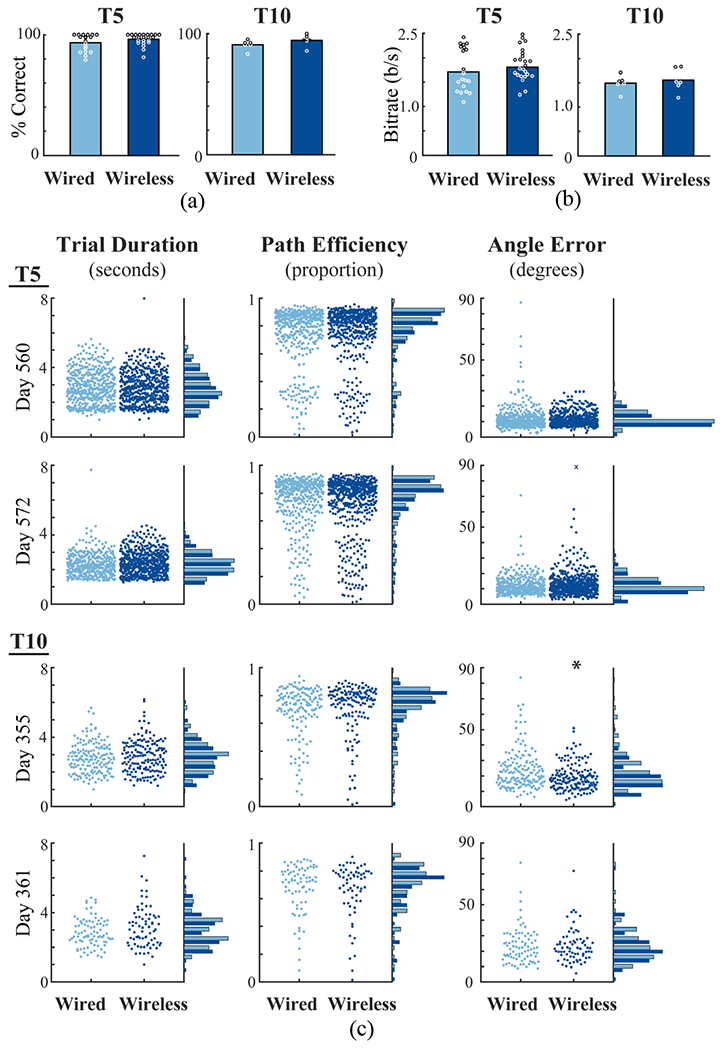
Metrics comparing closed-loop cursor control in wired (light blue) and wireless (dark blue) configurations. (**a**) Median target acquisition rates in wired and wireless conditions. Circles indicate the measure for each iteration of the Grid Task across two sessions for each participant (white and black used for contrast). (**b**) Bitrates in wired and wireless conditions (one measure for each Grid Task across two sessions for each participant). (**c**) Three metrics of cursor control over two days for each participant. Each point shows the metric computed for an individual trial (one target acquisition). Points are spread on each X-axis to reveal individual trials. Histograms on the right of each plot summarize wired and wireless performance. ‘x’ indicates an angle error exceeding 90 degrees. ‘*’ indicates significant difference (P<0.05).

The percentage of successful target selection was not significantly different between wired and wireless systems (Wilcoxon rank sum > 0.05) for either participant (Fig. 3a). T5 median success rate was 96.1% versus 97.9% in the cabled and wireless conditions, respectively. T10 achieved 91.6% and 94.8% in cabled and wireless configurations. Bitrates in the Grid Tasks were also not significantly different (Fig. 3b) between cabled and wireless systems for either participant (Wilcoxon rank sum > 0.05; median bitrates T5: 1.62 bps cabled and 1.81 bps wireless; T10: 1.51 bps for both systems).

These statistics provided one measure per task iteration (per block of trials). We also quantified cursor control at the resolution of individual trials (Fig. 3c). Across three metrics (Trial Duration, Path Efficiency, Angle Error), no significant differences were observed on either day with T5 or on trial day 361 with T10. Significant differences were observed only for T10 angle error on day 355 (Wilcoxon rank sum < 0.05). On this day, the median measure for angle error (20.2 degrees wired, 18.0 degrees wireless) were better with the wireless system. While the causes of this slight apparent superiority of the wireless system are not clear, the wired condition exhibited more outlier trials with high angle error on this day.

### C. Wireless iBCI Use of a Tablet Computer

T5 and T10 each used the wireless iBCI to achieve point-and-click control of a standard consumer Microsoft Surface 2-in-1 tablet computer running Windows 10 (Supplemental Material). On trial day 307, after completing an autocalibration sequence, T10 started several widely-used apps in sequence by selecting the corresponding app tile on the Windows start menu or desktop. He used Pandora, the NCAA basketball app, Skype, YouTube, Gmail and the Weather app (Supplemental Video). T5 used these same apps (excluding NCAA) on his trial day 588, including typing on the Windows on-screen keyboard to perform searches and compose messages in Gmail. T5 also used the on-screen keyboard to type spontaneous sentences, such as “Beata I understand that you may have a loaner cat.”, into Windows Notepad (Supplemental Video). Over 814 seconds (14.6 minutes) in the Notepad app, T5 typed 196 correct characters; the backspace key and any characters contributing to misspelling or that T5 subsequently deleted were included in the elapsed time but not in the correct character count. This yielded 13.4 correct characters per minute (ccpm) as a measure of free, spontaneous typing performance while using the wireless system with an onscreen keyboard.

### D. Wireless Acquisition During 24 Hours in the Home

In T10’s 24-hour study, synchronized neural data were wirelessly recorded from two intracortical arrays between 2:22 pm on trial day 349 and 2:22 pm on trial day 350. With pre- and post-session activities, total participant engagement time was 26 hours. Each array logged 500 GB of broadband data (1 TB for 2 arrays over 24 hours).

**Fig. 4.**
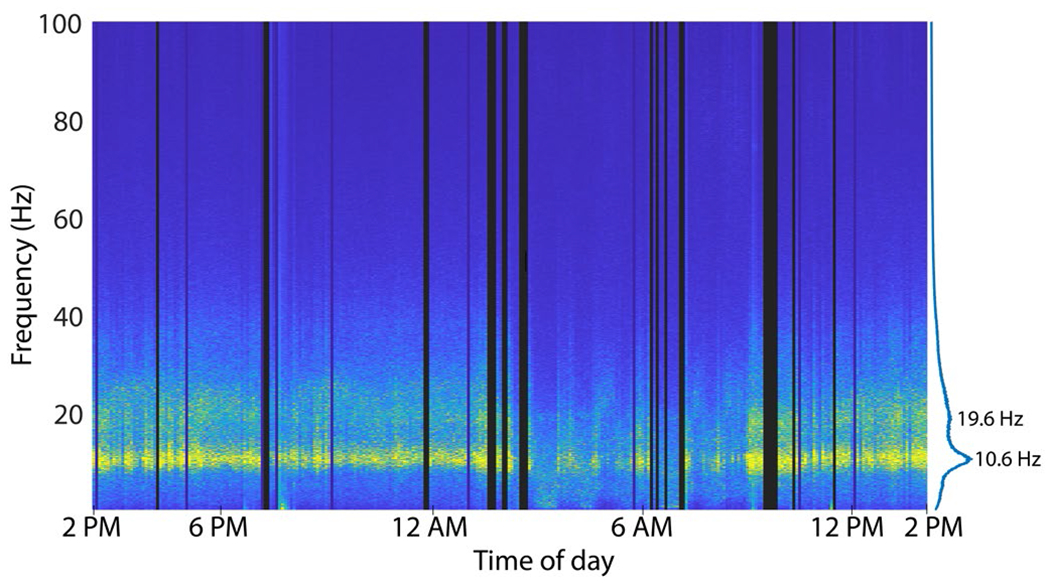
Spectral content of T10 neural data recorded continuously over 24 hours with the wireless system (posterior pedestal). X-axis indicates wall-clock time. Dark vertical bars reflect periods where data was not recorded (e.g., transmitters removed) or was severely disrupted (high frame loss). Peaks in the spectral power are noted on the right at 10.6 Hz and 19.6 Hz.

We examined the spectral content of the recorded signals for evidence of transmitted neural activity and/or recording artifacts. Spectral content of the recordings across the 24 hours was evaluated in contiguous 5-minute segments (Fig. 4). Prominent LFP activity was observed in bands centered at 10.6 Hz and 19.5 Hz. Clear changes in the spectral power were evident during the night and early morning (e.g., in the ~20-40 Hz band) corresponding to the period when T10 was observed to be sleeping (approximately 3 am to 9 am).

Over the 24 hours (1,440 min.), wireless recording was highly reliable. Wireless recording was uninterrupted throughout most of T10’s activities, including using a head tracking system to type emails and browse the web, having a phone conversation, watching TV, talking, eating, sleeping and more. However, “disruptions” in the form of unrecorded data or noise were observed in 35 of the 288 five-minute analysis segments (175 total disruption minutes). A review of the session logs found that the large majority of data disruptions (100 minutes) occurred when one or more caregivers were attending to T10 including rotating or shifting him in bed, suctioning, and other nursing care (Table 1). During these periods, caregivers worked in close proximity to the bed including standing directly between the transmitters and antennas for several minutes at a time. Data were recorded but exhibited packet loss which was sometimes accompanied by substantial noise at the moment when the signal was recovered. In other cases, data flow stopped entirely when transmitters were removed during battery replacement, bathing and dressing. Other disruptions were not related to the wireless components but rather resulted from technical glitches in the real-time data file storage process. Only 15 minutes of data disruption were not readily associated with noted activities; these occurred during sleep between 6:45AM and 7:15 AM.

### E. Benchtop Comparison of Cabled and Wireless Recordings

**Fig. 5.**
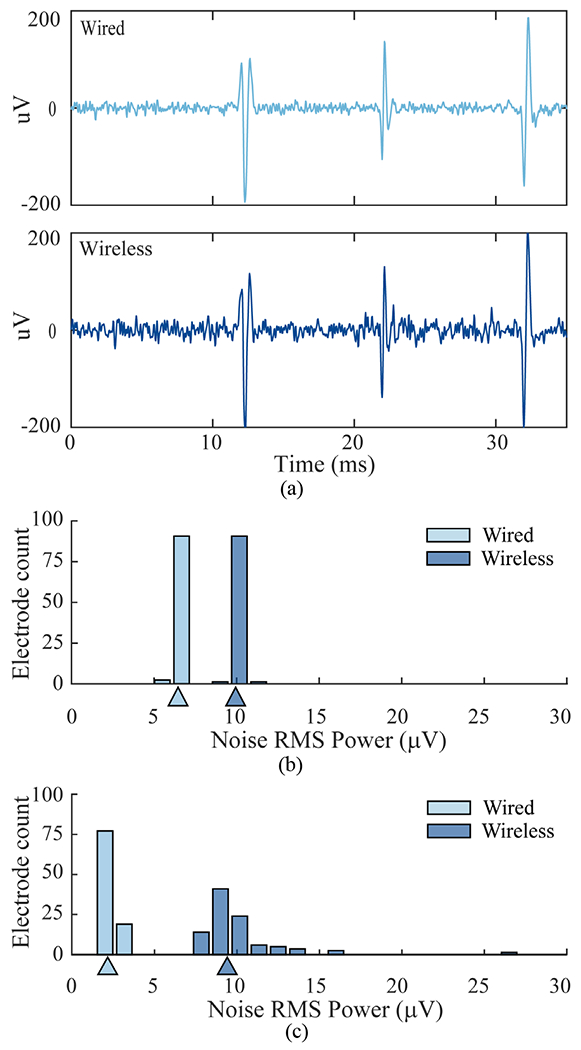
Comparison of wired and wireless recordings of simulated neural signals, (**a**) Waveforms of three different spiking neurons aligned from bandpass-filtered wired and wireless recordings, (**b**) Distribution of baseline noise in the spike-filtered data (250 Hz – 7.5 kHz) across all 96 channels. Noise was measured as RMS power of the residual signal after all spike events were removed. Triangles indicate median noise values, (**c**) Distribution of noise values in the field potential range (5 Hz – 250 Hz) computed for each channel after removing the 96-channel mean signal recorded in the wired condition.

To provide a baseline comparison of the two systems, we performed benchtop tests in which well-defined simulated neural signals from the Neural Signal Simulator (NSS) were recorded through the cabled and wireless pathways. The NSS generated continuous broadband data which superimposed noise, low-frequency oscillations and “action potentials” mimicking three spiking neurons with distinct waveform shapes. After applying a spike-band filter and aligning corresponding simulated spike waveforms, there was a close correspondence between the signals recorded with the two systems (Fig. 5a). We observed low overall noise levels in both conditions with a few microvolts more noise (RMS) with the wireless system in both the spike band (Fig. 5b; wired noise: 6.5 μV; wireless: 9.9 μV) and the LFP-filtered band, (Fig. 5c; wired: 2.2 μV; wireless: 9.4 μV). This noise metric, which quantified the difference between the recorded signal on each electrode relative to the mean (multi-electrode) signal in the wired condition (Supplemental Methods), revealed slightly higher variability of field potential recordings across channels in the wireless system. Below 5 Hz, the wireless system will exhibit greater signal attenuation than the wired system due to the wireless 1 Hz low-frequency cutoff (versus 0.3 Hz for the wired system). The BWD low-frequency cutoff can be set to other values during device assembly.

To further evaluate the fidelity of spike recording, we applied an offline automated unsupervised sorting algorithm to recover the waveform shapes and firing rates on all electrodes [30], The simulator’s three unique waveform shapes were all reliably recovered on all electrodes as indicated by equivalent waveform sorting templates in wired and wireless conditions. The equivalence of units recovered from wired and wireless data sets was evident when mean waveforms and their standard deviation were overlaid (Supplemental Fig. S2). The firing rates of all units on all 96 channels were identical (3.6 Hz) and matched the simulated rates, confirming that action potentials were recorded with the requisite fidelity.

### F. Wirelessly Recorded Human Intracortical Signals

**Fig. 6.**
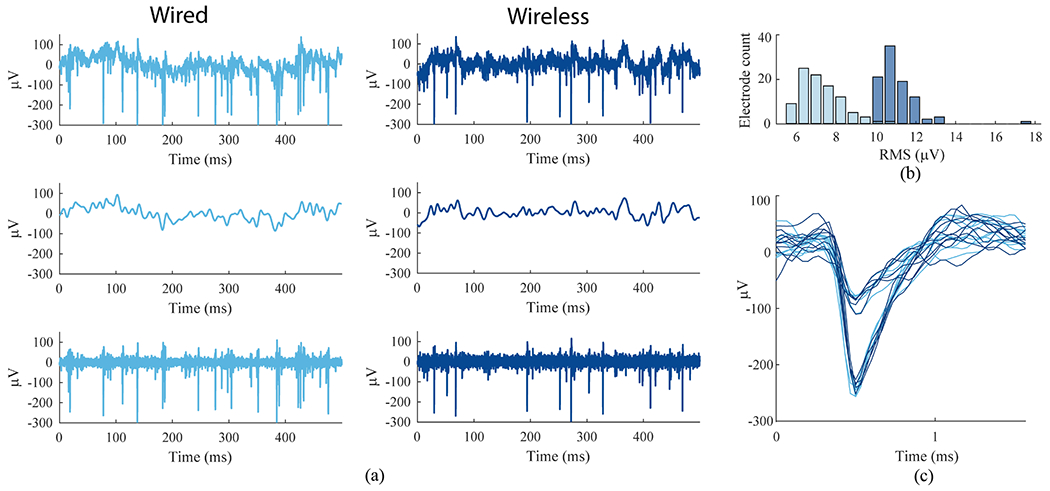
Human intracortical signals recorded in wired and wireless configurations in the home, (**a**) Comparison of recorded neural activity on one electrode (t10 trial day 361, e24 blocks 6, 7). Top: the “raw” unfiltered neural signal. Middle: low-pass filtered (100 Hz cutoff). Bottom: band pass filtered for spike extraction (250 Hz — 7.5 kHz). (**b**) Distribution of residual RMS amplitudes from all electrodes on one array after band-pass filtering and removing thresholded spikes for wired (light blue) and wireless (dark blue) recordings, (**c**) Sample waveforms from two units sorted from the same electrode shown in (a) and (b). Light blue (wired) and dark blue (wireless) waveforms show substantial similarity.

We compared human neural signals recorded with wired and wireless systems during all Grid Tasks. The raw (unfiltered), LFP-filtered, and spike-filtered signals were substantially similar across conditions (Fig. 6a). Noise in the spike-filtered intracortical data was slightly lower than observed with the simulator. Consistent with the NSS tests, noise in the spiking band was a few microvolts higher in the wireless condition (Fig. 6b). Spike waveforms were equivalent between the recording modalities as demonstrated by overlaying wired and wireless waveforms for putative single units (Fig. 6c). We extracted waveforms for spiking units on all electrodes, applying the same unsupervised algorithm and parameters to wired and wireless data recorded during Grid Tasks. Comparable single unit waveforms were extracted across most electrodes. However, several units were observed in only one condition or the other. These were usually, but not always, small amplitude units or waveforms that were inconsistently split into multiple units. Thus, the population of extracted units was not identical between wired and wireless data. However, we also observed similar variability between Grid Task blocks recorded with the cable alone. Given the fidelity of recording confirmed in our simulator tests, these discrepancies were most plausibly attributed to nonstationarities in the recorded intracortical signals [17] rather than failures in the wired or wireless systems.

### G. Wireless Packet Loss During In-Home iBCI Use

To quantify the incidence of data loss during use of the wireless system, we inspected all raw 30 kS/s data recorded from each participant’s two arrays during all Grid Tasks completed by T5 (24 blocks, 47.9 min) and T10 (6 blocks, 10.3 min) over 2 days each. A “packet drop” occurred when the sync word delimiting a 50 μs wireless data frame containing a single sample from all electrodes could not be recovered by the receiver, resulting (by design) in a repeat of all electrodes’ previous digital values being re-inserted into the receiver’s output stream.

The incidence of dropped packets varied across participants and pedestal position (Table II). For each participant, signal recovery was consistently more successful for one transmitter (anterior 3.3 GHz for T5, posterior 3.5 GHz for T10) than the other. For T5, packet drops were observed during 8 of 24 blocks from the anterior pedestal and during all 24 task blocks from the posterior pedestal; the proportion of dropped packets was overall very low (0.003% of all frames, anterior; 0.4163% of all frames, posterior). No packet drops were ever observed for T10’s posterior pedestal and rarely for the anterior pedestal (fewer than 5 total packet drops per block). In one block, however, T10’s anterior packet drops occurred with a drop rate of 4.69%. We attribute differences in the fraction of dropped packets among blocks to changes in orientation of the participant (transmitters) relative to the antennas. Although participants had limited movement, the four-antenna configurations that we used to simplify session setup in the homes may not have provided sufficient simultaneous spatial coverage for both directions of transmission from two BWDs.

It was noteworthy that during that 2-minute block with high packet drop on one transmitter, T10 nonetheless acquired 44 of 45 targets and recorded his highest bitrate in this study (1.79 bps). It is possible that signals from the second transmitter were sufficient to sustain adequate decoding performance (the second transmitter experienced no dropped packets during this block). The performance impact of packet drops may also relate to whether they are sufficiently sparse (distributed over time) to avoid extended periods of impoverished neural decoding. To quantify this, we computed the number of Severely Errored Seconds (SES), any non-overlapping 1-second period in which half or more of the data samples were lost. Although packet drops occurred, SES events were rarely observed, limited to T5’s posterior data stream in 5 blocks with a cumulative total of 10 seconds. During this study, a total of 6984 wireless data seconds (nearly 2 hours) were recorded during the Grid Task assessments cumulatively across both participants and both arrays; only 10 seconds of these exhibited SES.

### H. Bit Level Analysis of Wireless Data

We examined the raw data files for any evidence of bit-level anomalies in the 30kS/s data upsampled from each transmitter. These analyses excluded epochs with signal anomalies attributed to dropped packets (analyzed above). The wireless data exhibited occasional instances of large instantaneous voltage discontinuities. These were biologically implausible voltage differences between consecutive 33 μs samples caused by bit flips among the most significant bits (MSBs) of a digital sample. The corrupt voltage value lasted only one sample (or two samples when an upsample repeat followed) then returned to baseline in a single step. These fast single-sample slew rates were sometimes observed among the lower bits as well, and it seems probable that lower bits were equally prone to flipping but the corresponding small voltage fluctuations would generally have been indistinguishable from baseline activity (and, presumably, functionally inconsequential). The incidence of MSB errors was inconsistent across channels and across blocks. In any given recording, some channels could be recorded error free while others exhibited rare, or more frequent, bit errors. Nonetheless, bit errors were observed in every wireless recording (at a rate of 2.04E-5 to 9.26E-5 per sample throughout the 30 Grid Task blocks). Another form of bit flip persisted for three or more samples and recurred multiple times over a period of tens or hundreds of milliseconds with seemingly valid sample data in between. These periods of recurring MSB errors, which we termed “digital noise”, presented as noisy epochs in the voltage data. Digital noise events were rare in our Grid Task data but more likely in the 24-hour data. In general, we observed a higher incidence of all bit errors including digital noise after periods of packet drops and in association with putative antenna SIMO switching events. Bit errors also appeared to be associated with the upsampling logic (see below). Bit errors could have been introduced at various stages of the wireless system such as the BWD analog-to-digital converter, the wireless link, SIMO antenna switching, and the clock domain boundary in the upsampling process. The data collected here did not allow concrete identification of the source(s) of these errors. MSB errors were not observed in the cable-recorded data.

Because MSB errors were identified in our preliminary work, this study applied an algorithm to detect MSB-corrupted samples (briefly, any voltage shift 500 μV or greater within a single sample, i.e., a 15 μV/μs slew rate) and replace each with its preceding good sample on that electrode. In this way, the impact of flip-bit errors was minimized in real-time, prior to feature extraction and decoding, and in offline analyses.

As expected by design, the recorded wireless data contained a large proportion of 33 μs data frames that contained perfect copies of the prior samples on all 96 electrodes. These “upsample repeats” resulted directly from the sample-and-hold logic that upsampled the 20 kS/s BWD data to the NSP 30 kS/s format. Unexpectedly, one or more bits in the upsample repeat frame occasionally differed from their original value. Although these anomalies needed to be accounted for during analysis, they did not otherwise impact wireless performance.

## IV. Discussion

This study demonstrates the first human use of a broadband wireless intracortical BCI. Two participants directed computer cursor movements and click decoded by an iBCI that acquired and wirelessly transmitted (previously prototyped as [25]) broadband neural activity from 192 chronically implanted microelectrodes. Across multiple signal and iBCI performance metrics, the wireless solution proved to be a thorough replacement for the cabled connection currently used in chronic human iBCIs studies and fundamental non-human primate research. Raw neural data were processed into spike rates and LFP power in the spike band which were decoded together to yield precise iBCI cursor control. Using the wireless iBCI, study participants achieved communication bitrates in a Grid Task equivalent to the wired system. Self-paced cursor control enabled both participants to browse the web and complete other tablet activities, and one participant used the wireless interface for free typing. The wireless technology also enabled continuous recording of intracortical broadband field potentials and spiking activity from one participant over 24 hours in his home. Untethered recording of intracortical signals and in-home iBCI use are major steps toward an on-demand iBCI to provide independent digital communication and computer access for people with severe motor impairment.

Evaluation of the raw neural signals and spike waveforms found that signal processing differences in the cabled and wireless pathways had negligible impact on the quality of the recorded neural signals. A small increase in baseline noise with the wireless system was detectable but had no functional impact. The wireless system did exhibit bit errors which we were able to mitigate with real-time algorithms. It proved possible to record broadband intracortical signals in the home without packet drops, as demonstrated by many data blocks in which one or the other transmitter exhibited zero packet drops. Packet drops that did occur were exceedingly rare, indicating the fundamental reliability of the wireless system. Because wireless signal recovery is sensitive to plane of transmitter orientation and distance relative to the position and polarization orientation of the receiving antennas, we anticipate that packet drops could be reduced by more optimal placement of the antennas or by using the full complement of antennas supported by the receivers. Critically, the fidelity of the broadband neural signals and iBCI performance were robust to the low incidence of packet drops that was observed in most blocks. During 24-hour in-home recording we found that volitional head movements and even body rotation by nursing staff generally did not interrupt the wireless signal. However, someone standing or working close to the participant’s head could block transmission entirely, resulting in data loss or “digital noise” during some periods of nursing care. For this study, we elected not to modify users’ homes or wheelchair to accommodate more robustly-placed or permanent antennas. However, these findings motivate further investigation of optimal antenna placement in the home.

### A. Design trade-offs

The BWD system deployed in this study prioritized uncompromising signal fidelity and high electrode count to facilitate both iBCI applications and fundamental human electrophysiology research. However, as the field progresses toward higher and higher electrode counts, technologies for broadband recording become increasingly challenging and alternative, lower-bandwidth signal acquisition approaches are being considered. Design specifications for a wireless targeted BCI multielectrode acquisition system could be simplified by limiting signal acquisition to spiking events (with or without sampled spike waveforms) without broadband data. Although sorted single-unit activity and threshold crossings contain distinct information [31], [32], decoding thresholded multiunit activity rather than sorted units can enable precise iBCI control in monkeys [33–35] and in our own BrainGate participants with tetraplegia [4], [7]. Decoders built on multiunit thresholds can also be maintained over long periods [4], [7], [36] and population dynamics may be estimated from multiunit threshold crossings without spike sorting [37]. An analysis of iBCI decoding in monkeys and humans proposes that digital sampling at 1 kHz should be sufficient for iBCI applications [21]. These approaches could reduce the wireless bandwidth by roughly an order of magnitude, thereby simplifying the engineering design constraints for a fully implanted wireless iBCI or, alternatively, freeing bandwidth to record from thousands of electrode contacts [38–43].

Despite the broadband design requirement, the BWD design also prioritized low power for long battery life to facilitate users with disability using the system throughout their day. The low power design also translates into fewer inductive charging periods for the fully-implanted version of the BWD (currently in pre-clinical testing). There are several clear trade-offs, however, and comparison to other systems is informative. A pedestal-mounted wireless interface with a commercial WiFi chipset has been used in an unconstrained non-human primate [44]. While similar to the BWD in terms of signal sampling and channel count, the package was relatively large, had high power consumption and incorporated a cooling fan. A subsequent commercial device modified from our prototype, Cereplex-W (Blackrock Microsystems) prioritized slightly lower noise, 16-bit 30kS/s sampling, error correction in the wireless link, and more powerful transmission — with the tradeoff of requiring much higher power consumption than the BWD and therefore much shorter operation per battery charge.

Other broadband wireless systems for multielectrode research have been demonstrated in freely moving primates [44–50] and a growing number of commercial alternatives support wireless intracortical recording for animal research. To our knowledge none are yet approved for human use, but several designs have a level of integration and other design tradeoffs that could be consistent with future translation. For any of these systems, engineering and translational challenges toward chronic human use include pairing the wireless components to intracranial electrodes suitable for long-term implantation in people, a small and lightweight package, high data rates (and/or on-board processing) across many electrodes, long run time (low power consumption), successful safety testing and completion of regulatory activities.

### B. Comparison to Other Wireless Implanted BCIs

An early wireless broadband BCI demonstrated the ability for a person with ALS to toggle a binary signal from a single intracortical electrode [51]. One early ECoG closed-loop system with 32 subdural electrodes enabled an individual with tetraplegia [52] to move a cursor to targets by associating four different visualized movements with four cursor directions. Although neither wireless nor chronic, this system demonstrated a degree of multidimensional cursor control based on decoded intracranial signals. More recently, a 128-electrode chronically-implanted wired ECoG array enabled reliable point-and-click by an individual with paralysis. The participant achieved > 85% accuracy and some bitrates over 0.8 on a 4×4 grid task [53]. Notably, performance with that ECoG BCI remained relatively stable over weeks without decoder recalibration, similar to recent stabilization reported with Blackrock microelectrode arrays in nonhuman primates [54].

A novel approach to intracranial recording, via electrodes attached to an endovascular stent [55], is now in clinical feasibility trials. Such systems, while low bandwidth, may represent an effective solution for some individuals with severe disability [56].

Recently, deep brain stimulators (DBS) have served as a convenient, clinically accepted platform upon which fully implanted wireless BCIs have been developed [57–59]. A wireless BCI, configured from a bi-directional fully implanted DBS device (Activa PC+S, Medtronic, Inc.) was used to sample field potential data (0.8 kS/s) from a bipolar pair of chronic subdural ECoG electrodes in a person with ALS [58]. The wireless link from the subclavicular transmitter (11.7 kbps) was more than 1,000x lower bandwidth than one BWD; nevertheless, this enabled the individual to use the BCI to make click selections in a commercial scanning letter interface. The research participant spelled 44 prompted words at a mean rate of 1.15 correct characters per minute (without letter prediction). Despite the slow communication rate, the participant reported satisfaction with the system. In another study, a fully-implanted PC+S system was evaluated for subdural ECoG recording of motor-related potentials from five patients with Parkinson’s disease over the span of a year [59]. Up to 9 minutes of data from two subdural sites could be recorded on the device before being downloaded wirelessly for offline analysis. Although no closed-loop study was attempted, movement-related changes in beta (and sometimes gamma) bands were observed in the recorded data, possibly relevant to a future closed-loop BCI implementation. W-HERBS is an ECoG BCI system being developed to record up to 128 subdural electrodes through a cable tunneled from the head to an inductively rechargeable implanted abdominal device with WiFi capability [60]. In a bench evaluation, that system digitized sine waves at 1 kS/s per channel and transmitted data wirelessly with 250 ms latency to a data storage workstation. W-HERBS has been advancing over many years but has not yet been tested *in-vivo.* Another ECoG study evaluated a bench prototype form of a future implantable abdominal device cabled to a 32-channel integrated front-end ADC (0.5 kS/s) [61]. Unlike W-HERBS, however, that abdominal device design incorporated two low-power processors so that ECoG signals could be decoded onboard and state estimates transmitted out every 750 ms. In a bedside test with a study participant implanted with electrode strips for seizure monitoring, that externalized BCI system decoded move (actual hand grasp) versus idle (hand relax) with 87% accuracy over 12.5 minutes. Neither of these abdominal systems has been evaluated *in-vivo*. In contrast, the epidural WIMAGINE® ECoG neural interface (Clinatec, Grenoble, FR), was recently demonstrated in an individual with SCI [62]. Two devices were placed over bilateral motor cortical areas for chronic epidural recording. The wireless data link supported 586 samples/second x 32 contacts using external antennas held near the implants by a custom helmet. Using the BCI to activate a neural on/off switch, the participant was able to initiate walking movements of an avatar and an exoskeleton. The participant also commanded continuous control of bilateral limb movements of the exoskeleton and was able to steer a powered wheelchair. We have not yet evaluated the current wireless system relative to cabled intracortical control of robotic, prosthetic, or functional electrical stimulation applications [1–3], [11], [12]. However, the higher electrode count and 100x data rates demonstrated here provide the potential for more dexterous low-latency wireless motor prostheses. Whether ECoG BCIs can approach the communication performance demonstrated here remains to be determined.

While this broadband BCI provided a high-performance interface, cost of the various components currently used to support this performance relative to alternative, lower-bandwidth implanted systems will be important to consider as the field advances.

### C. Leveraging Broadband Wireless Recording

While the appropriate recording fidelity for stable, high-performance intracortical BCIs in people remains an active area of research, recent advances in high-performance iBCIs have leveraged broadband recording by decoding discrete spike rates combined with power in high-frequency local field potentials (HF-LFPs) up to 450 Hz [7], [12], [16] or 5 kHz [4], [28]. This “hybrid” decoding has contributed to stable high performance iBCI cursor control [4], [7] and rapid decoder calibration [28]. This and other iBCI studies have incorporated common average referencing computed across a subset of electrodes at high sample rate (e.g., 500 Hz [16]; 15 kHz [4], [7], [28]) to reduce noise prior to band pass filtering that could otherwise alias noise into the features computed for decoding. This technique computes a common signal from a subset of channels in real-time and applies it to all electrodes prior to computing neural features for decoding. Here, threshold crossing spike rates were computed after first applying a non-causal (bidirectional two-pass) bandpass filter with a 5 kHz upper cutoff frequency that has been shown to measurably improve decoding and BCI cursor performance relative to simple causal thresholding [63]. While we have not yet rigorously established a precise bandwidth that is necessary or sufficient for iBCI performance and stability for people with tetraplegia, these current practices provide a possible reference point for future optimized wireless BCIs (see also [22]).

### D. Future Work

One advantage of recent ECoG or stent-based BCIs relative to the external wireless system in the current study is that they are fully implanted with no percutaneous connections. A fully-implantable version of the BWD [26], [27], modified with a titanium package and inductive power, has been tested in animals and is being readied for regulatory testing prior to potential deployment in human clinical trials. To that end, the wireless device used in this study incorporates major elements of the fully implanted design and represents an externally-mounted prototype of those core technologies. We have also demonstrated a prototype battery-powered mobile embedded decoding system [64] that integrates with both the external and fully implanted transmitters and is readily interfaced with other future electrode technologies with hundreds or thousands of contacts.

The use of the unlicensed bands in the 3.3 to 3.8 GHz range was purposeful, but will require ongoing appraisal. Critically, the device is very low power and designed only for short-range transmission (a few meters). RF testing measured peak electric fields well below the FCC’s limits for unregulated bands in this frequency region. While this radio technology has been permitted to be used within the BrainGate study, further regulatory or engineering considerations may be required to advance the technology beyond the BrainGate trial or to accommodate regulations outside the U.S.

Intermittent wireless signal dropouts had limited impact on BCI performance in this study’s point-and-click tasks. However, performance degraded when the signal path was overtly blocked for a period of time. Fortunately, signal dropout can be readily detected by software in real-time. Nevertheless, wide-ranging independent use at home or as part of a fully mobile system could reveal additional signal dropout as participants navigate through the home in a wheelchair or move in and out of antenna range. Future work will develop real-time signal evaluation algorithms and a user interface system to notify users and caregivers about the quality of received neural signals and the corresponding instantaneous reliability of the BCI system. This will enable users or caregivers, for example, to reposition the wheelchair for better reception.

When integrated with stimulation capabilities, human wireless intracortical systems will offer new opportunities beyond iBCI [65] in neural sensing and closed-loop neuromodulation, including the treatment of seizures and neuropsychiatric disease [66–68]. Limitations of this current generation BWD and its implantable sibling are their unidirectional communication; neither stimulation nor impedance testing are currently possible. ASIC chips are being actively developed to introduce stimulation and impedance spectroscopy to future generations of these wireless devices.

Looking to the future, features of neural activity beyond spiking (e.g., high-frequency field potential oscillations, field-field phase coherence, spike-field relationships) remain to be fully explored in people and could one day be leveraged to enhance iBCI capabilities, performance and robustness. High resolution intracortical recording – currently only feasible as part of clinical research endeavors – will facilitate continued investigation of aspects of neural activity that may be clinically useful. We anticipate significant advances and paradigm shifts in neural signal processing, decoding algorithms, and control frameworks that will advance neural decoding for effective iBCI and neuromodulation applications. When, how and if different groups (academic, industry, etc.) should diverge in their efforts to develop better and clinically viable iBCI systems is a discussion central to iBCIs’ successful translation.

## V. Conclusion

This study reports important progress in the clinical translation of an iBCI toward a future assistive medical device for individuals with paralysis. We demonstrate the first high-resolution broadband recording from multiple implanted microelectrode arrays using a wireless iBCI in human subjects. Results with two individuals with tetraplegia demonstrate the viability of a wireless iBCI for real-time control of a point-and-select interface. Characteristics of the BWD recordings were highly comparable to those of wired recordings. This broadband wireless system also enables ongoing fundamental research into cortical processing during every day human behavior to inform future neuroscience and BCI advancements. This work overcomes several former barriers to in-home mobile independent use of a promising assistive technology to restore communication and digital access for individuals with severe speech and/or motor impairments.

## Supplementary Material

Simeral - Supplemental Material

Supplemental Video 1 (T5)

Supplemental Video 2 (T10)

## Figures and Tables

**Table I.** Events associated with disrupted wireless signals over 24 hours

Total Minutes	Activity
100	Caregivers attending T10 (rotate in bed, suction)
25	Bathing, dressing (transmitters removed)
15	Unexplained signal noise during sleep
15	Technical errors in real-time file storage system
10	Battery changes
10	File breaks before/after cursor sessions

**Table II.** Incidence of wireless data packet drops during Grid Tasks for anterior (Ant) and posterior (Pos) data streams.

ID	# Task Blocks	Total Time (Min)	# Blocks with Dropped Packets		Dropped Packets (% of All Data)		# of Blocks with SES		Total SES Time (sec)	
			Ant	Pos	Ant	Pos	Ant	Pos	Ant	Pos
T5	24	47.9	8	24	0.003	0.416	0	5	0	10
T10	6	10.3	6	0	1.004	0	0	0	0	0
