# Supplementary material for: Home Use of a Percutaneous Wireless Intracortical Brain-Computer Interface by Individuals With Tetraplegia
Source: IEEE Trans Biomed Eng. Author manuscript; Available in PMC 2021 Jul 1. (PMC8218873; doi:10.1109/TBME.2021.3069119)
Supplement: Simeral - Supplemental Material [file NIHMS1706467-supplement-Simeral_-_Supplemental_Material.pdf]

Supplementary material for:

Home Use of a Percutaneous Wireless Intracortical Brain-Computer Interface by Individuals With Tetraplegia

John D. Simeral, Thomas Hosman, Jad Saab, Sharlene N. Flesher, Marco Vilela, Brian Franco, Jessica Kelemen, David M. Brandman, John G. Ciancibello, Paymon G. Rezaii, Emad N. Eskandar, David M. Rosler, Krishna V. Shenoy, Jaimie M. Henderson, Arto V. Nurmikko, and Leigh R. Hochberg

I. Contents

Supplemental Methods, Figures and Tables 1
Table SI. Major specifications of the BWD transmitter 2
Figure S1. iBCI-enabled typing to search in the YouTube app 3
Table SII. Tablet apps activities tested by T5 and T10 4
Figure S2. Waveforms of simulated action potentials recorded in wired and wireless conditions 5
Supplemental Videos 6
References (for Supplementary Material) 6

II. Supplemental Methods. Figures and Tables

A. Standard Cabled iBCI System

The cabled BrainGate iBCI used commercial hardware and software (Blackrock Microsystems) to acquire and record neural signals. This system included a NeuroPort Patient Cable connecting each percutaneous head-mounted pedestal to a Front End Amplifier which digitized signals on each of 96 microelectrodes (30 kS/s, 16 bits per sample). The continuous serial stream of digital samples was relayed over fiber optic cable to a Neural Signal Processor (NSP) where they were timestamped and sent out as UDP packets on a private local area network. These "raw" data were stored (without software filtering or down-sampling) by Blackrock's Central Suite software for offline analysis. Because participants in this study each had two arrays, the home iBCI included two parallel 96-channel acquisition systems that were time-locked by a sync cable linking the two NSPs.

The raw data packets were also delivered in real-time to downstream Assistive Technology computers for signal processing, neural feature extraction and decoding. The Assistive Technology system included a Super Logics x86 computer (Natick, MA) running the xPC Target real-time operating system (The Mathworks, Natick, MA) and custom signal processing and decoding algorithms running in Simulink. Decoded cursor commands were sent from Simulink to a Dell laptop (T5) or a Microsoft Surface tablet (T10) over wired Ethernet, wireless Ethernet (through a 5GHz WiFi router), or Bluetooth. Bluetooth communication employed our previously developed circuit board dongle based on BlueSMiRF technology (SparkFun Electronics, Boulder, CO) that plugged into the xPC Target computer and presented BrainGate decoder outputs as standard wireless mouse point and click commands [8] [69].

B. Wireless iBCI System

The Wireless Receiver supported up to 8 antenna inputs in a Switched Diversity Single-Input Multiple Output (SIMO) architecture for improved reception. SIMO provides robust recovery of a single communication link (one BWD's continuous transmission) by allowing multiple antennas (e.g., in a home environment) where the signal can

be retrieved (i.e., multiple outputs of the communication link). The receiver considered a data frame to be valid on any antenna input when the expected terminating sync word was detected ("signal lock") which was indicated by a blue LED on the front panel. The sample data in each frame did not include error checking, so it was possible for bit errors to occur in the data even when the sync word was detected. SIMO was used to provide a degree of data checking: when two or more receiver channels reported lock, the data output by the receiver was taken from a channel whose contents exactly matched those of another locked channel.

After preliminary wireless reception testing in the home of T10, we elected to reduce the physical footprint of the wireless system by using four (rather than 8) antennas mounted above and/or behind the user, 1-2 m from the pedestal-mounted transmitters. A tripod stand behind his bed allowed four antennas to be positioned near the head and adjusted to achieve effective angle and polarization orientations. For T5, two antennas were positioned on tables near his wheelchair and two at the ceiling slightly behind the wheelchair (main text, Fig. 2d). During setup for each recording session, we ensured that LEDs on each receiver consistently indicated signal "lock" for each antenna input but did not attempt to further optimize the placement of antennas.

Prior to the first study session with T10, we performed an in-home spectral sweep for potential radio frequency (RF) interference with a microwave spectrum analyzer (Anritsu). No detectable sources of interference in the 3.0 – 4.0 GHz band used by the BWD were found, despite numerous in-home appliances, medical devices, and a nearby line-of-sight commercial radio tower.

C. Pedestal-Mounted Wireless Transmitter (BWD)

Key BWD device specifications are summarized in **Table SI**.

Table SI. Major specifications of the BWD transmitter

Characteristic	Value
Number of channels	96 neural recording (+ 4 other)
Sampling rate per channel	20 kS/s
Bit resolution	12 bits/Sample
Bandwidth	1.0 Hz – 7.8 kHz
Transmission frequency	3.3 GHz or 3.5 GHz
Transmission modulation	OOK
Total neural data rate	24 Mbps
Transmission rate	48 Mbps (Manchester encoded)
Transmission power	-1.6 dBm
Size (including battery)	52 x 44 x 30 (L x W x H, mm)
Weight	46.1g with battery
Total operating power	51 mW (max) @ 3V
Power source, battery life	Li-$SOCl_2$ battery, 3.6 V, 1.2 Ah, > 35 h

D. Neural Decoding Methods

For iBCI cursor tasks, a Kalman filter decoder and a linear discriminant classifier were used to estimate continuous 2-D cursor velocity and click state, respectively. After upsampling in the wireless path (described above), data processing and decoding algorithms were identical for cabled and wireless conditions as described next. The signal processing and decoding algorithms used here have been described in detail previously [4]. Briefly, signals were downsampled to 15 kS/s followed by common average referencing. For decoding, two neural features were then computed for each electrode: spike rates and spike power. Spike rates were computed from thresholded spikes. Spike power was estimated as the power in the high-frequency local field potential (HF-LFP). For both features, a band pass filter was first applied (250 Hz - 5 kHz, 8th order IIR Butterworth). Spike rates were then computed by re-filtering the band-passed data in reverse to provide net zero-phase shift [63] before applying a spike threshold at -3.5 standard deviations (computed for each channel from prior within-day blocks of data). Spike power was computed by squaring and averaging the band-passed signal. Spike power and thresholded spike rate features were z-score normalized using each feature's mean value and standard deviation calculated from a previous data block. All neural features, cursor velocity, and state estimates were updated every 20 ms.

E. Grid Task Assessment of iBCI Cursor Control

Decoder performance in wired and wireless conditions was assessed on a series of Grid Tasks (2 minutes each) in which participants moved the cursor to the indicated target and then dwelled on the target to select it. The Grid Task consisted of a *6* x *6* grid of adjacent squares in which all points were selectable as described previously [1] [7] [29]. Dwelling on any target for 1 second selected it (resulting in a correct or error selection) after which the next target was shown. Selections continued uninterrupted until the Grid Task ended. If no target was selected within 8 seconds then a timeout resulted in an error trial and the next target was presented. The Grid Task was repeated several times with brief pauses in between. Task performance was assessed for each 2-minute task in terms of Percent Correct target acquisitions (hit rate) and ‘achieved bitrate’ quantifying information transfer (bits per second) as reported previously for this task [70]. Briefly, bitrate B is calculated as

$$B = \frac{\log_2(N)\max(S_c - S_i, 0)}{\mathrm{t}}$$

where N is the total number of selectable targets on the screen, S_c is the number of correct selections, S_i is the number of incorrect selections, and t is the total elapsed task time.

Metrics of cursor control included Trial Duration (average time to acquire targets including successful dwell time and the timeout duration for error trials, in seconds), Path Efficiency (ratio of actual trajectory length from starting point to the target relative to the straight-line path), and Angle Error (the mean angle between instantaneous trajectory and the direct-to-target trajectory, in degrees).

F. Wireless iBCI Use of a Tablet Computer

To evaluate whether the wireless neural interface system could enable individuals with paralysis to achieve reliable point-and-click control of a computer in their homes, T5 and T10 used the wireless iBCI for point-and-click control of an unmodified consumer mobile device (Dell laptop or Microsoft Surface Pro tablet) for web browsing, use of consumer applications, and typing communication. Cursor control was enabled using our standard automated decoder calibration, then CNRAs placed the tablet comfortably in front of the participant and routed the iBCI decoded cursor and click commands to the tablet [8]. Participants used the tablets in Microsoft Windows 10 “tablet” mode to navigate the desktop and to start and use different programs of interest to them (**Fig. S1, Table SII**). Additionally, T5 was asked to complete several blocks of typing using Windows Notepad to provide an estimate of typing performance. During these blocks, T5 used iBCI-enabled point-and-click to select characters and punctuation in the Windows native on-screen keyboard to type spontaneous sentences into the Notepad app and to select the backspace key to correct any mistyped characters **Supplemental Video 1**). Although the native onscreen keyboard offered word prediction, T5 did not use these but rather selected all letters, capitalization (“shift” function), spaces and punctuation explicitly. We computed correct characters per minute (ccpm) across all typing blocks as the number of appropriate character selections including spaces and punctuation (excluding characters resulting in misspelling, “shift” selected for capital letters, and delete or backspace key selections) divided by the total elapsed time. Detailed analysis of application use and typing rates were performed through offline analysis of screen capture recordings.

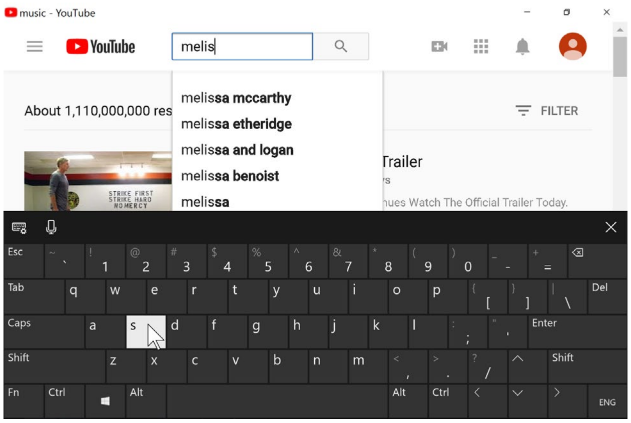

Fig. S1. Screen-captured image of T5’s tablet while he used the iBCI for point-and-click text entry with the built-in on-screen keyboard (in this instance to search a web site for music).

Table SII. Tablet apps used by T10 and T5 during wireless point-and-click

App	Activity
Pandora	Click to start Pandora in a web browser, select an artist, click the play button, select the "Thumbs Up" icon, click Pause, return to desktop
NCAA App	Select app tile on the desktop, select a game "recap" icon, then select the game video to play (T10 only)
Skype	Select the Skype icon in the start menu, select a contact and start a voice call, talk for 90 s
YouTube	Start app from the start menu, select on-screen keyboard icon, type search term, select a video, select "full-screen" icon, click back to desktop
Gmail	Click on Inbox icon, select a message and read it
Weather App	Click on Inbox icon, select a message and read it
Notepad	Type spontaneous phrases using the built-in Windows on-screen keyboard. (T5 only)

G. 24-Hour Wireless Recording

T10 completed an overnight in-home study to collect nearly continuous intracortical data over a 24-hour period. The study began with a typical CNRA-administered BrainGate research session in which data were collected during point-and-click cursor calibration and performance assessment. A similar research epoch was completed the next day to end the 24-hour period. Between these administered research periods, the wireless system was configured to continuously record and store raw neural data for offline analysis. For this first-ever overnight recording, two CNRAs maintained continuous monitoring on a rotating schedule. During this time, T10 remained in bed for reasons independent of this study, but he engaged in his typical daily activities such as eating, watching television, conversing by phone, direct family interactions, sleeping, etc. He received his regular nursing care including rotations in bed every few hours. Because RF pathway obstructions were anticipated as caregivers stood or moved between the transmitter and antennas, and because body rotations could alter orientation of the two transmitters relative to each antenna, a second pole of 4 antennas was added for this overnight study (increasing the number of antennas from 4 to 8). Based on laboratory testing, the battery in each transmitter was proactively replaced every 10 hours to avoid any possibility of unanticipated signal loss due to battery drain. (The wireless transmitter was designed to enable days-long continuous use per battery [24]; after this study, a correction to the BWD design schematic restored it to greater than 35 hours). A video camera captured snapshots of the room once per minute (with participant permission) to document activity. File recording software (Cerebus Central File Storage App, Blackrock Microsystems) automatically split neural data into contiguous files (with no sample loss) to limit the size of individual files.

Offline, integrity of the recorded data was analyzed in 5-minute segments contiguously across the 24-hour period (288 segments for each array). For visualization, the spectral content was computed from 0.5 Hz to 100 Hz in each segment. A "disrupted" segment was one with missing recorded data (e.g., brief stops between CNRA sessions and overnight recording), dropped packets, or pervasive noise (identified as segments in which the summed spectral power from 40 Hz to 100 Hz exceeded the 90th percentile of the entire 24-hour period).

H. Comparing Cabled and Wireless Signal Fidelity

We conducted a series of A/B experiments to more directly compare the fidelity of broadband recordings using the standard 96-channel NeuroPort Patient Cable "reference system" and the 96-channel wireless system. We compared noise, bandpass filtered signals, sorted spike waveforms and unit firing rates. These tests were performed with simulated signals in the lab and with intracortical signals recorded from participants at home.

Bench Measurements. An initial baseline comparison of signal fidelity was performed using a single NeuroPort Patient Cable or BWD transmitter to record well-defined signals from a Neural Signal Simulator (NSS, Blackrock Microsystems). Four antennas were placed within three meters and direct line-of-sight of the wireless transmitter to reduce the possibility of wireless data drops that could confound these baseline evaluations. The neural signal simulator provided continuous, well-defined patterns of voltages simulating slow local field potential oscillations (1 Hz, 3 Hz, and 9 Hz), three different repeating single-unit action potential waveforms on each channel, and baseline

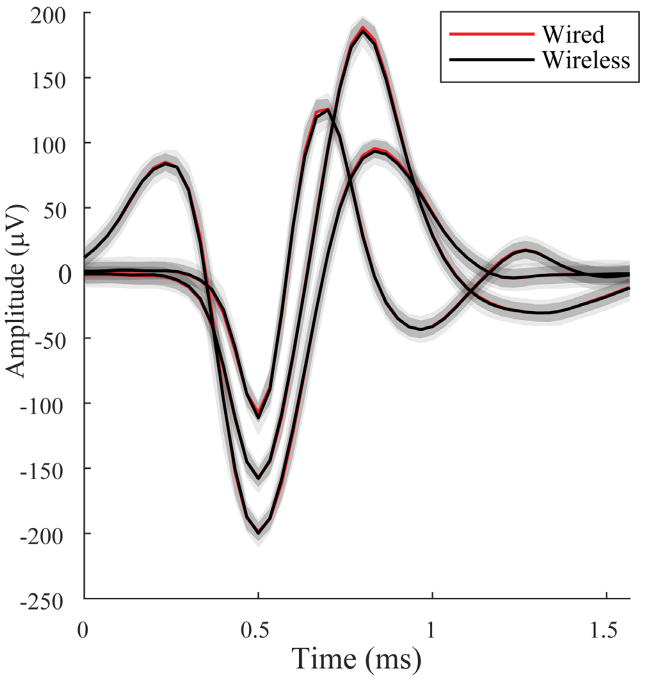

Fig. S2. Mean waveforms of the three simulated neurons as extracted from one representative electrode recorded by the cabled (red) and wireless (black) systems. Each unit shows mean (red or black line) +/- s.d. (gray bands) waveform averaged over a random selection of spikes.

noise. Several blocks of broadband data (~5 minutes each) were recorded in consecutive wired and wireless configurations and then analyzed offline. Because the signal simulator generated deterministic spiking patterns, it was possible to precisely align and compare the continuous raw recorded signals and spikes (waveforms) across conditions. To objectively compare spiking units without operator bias [71], we first applied unsupervised automated spike-sorting to each data set using a previously reported algorithm [30] to recover the waveform shapes and firing rates on all electrodes. We then compared the resulting sorted unit waveform shapes, spike rates, and noise measures (main text, Fig. 5). The simulator's three unique waveform shapes were all reliably recovered on all electrodes as indicated by equivalent waveform sorting templates in wired and wireless conditions. The firing rates of all sorted units on all 96 channels were identical (3.6 Hz) and matched the simulated rates, confirming that action potentials were recorded with the requisite fidelity. The equivalence of units recovered from wired and wireless data sets was evident when mean waveforms and their standard deviation were overlaid (**Fig. S2**).

We also quantified the baseline noise in the spike-filtered data on each channel as the power (RMS) of the residual after removing all samples in each spike waveform. We quantified noise in the LFP-filtered data (5 Hz – 250 Hz) on each channel as the power (RMS) of the residual after subtracting the mean LFP-filtered data recorded in the wired condition (averaged across all channels). This provided a measure of how much the recording on each channel in each condition varied from the wired reference standard. Those results were reported in the main text (Results, section E and in Fig. 5).

Intracortical Signals. Head-to-head comparisons evaluated data recorded with T5 and T10 during the closed-loop point-and-click Grid Tasks recorded in multiple interleaved cabled (A) and wireless (B) data blocks within each assessment day. A/B studies were repeated in a different order on separate days with both participants. As with bench measurements, comparisons of recorded spiking activity were made after automated spike sorting.

Calibrating Absolute Gains. To compare signal amplitudes across wired and wireless conditions, we applied an empirical gain adjustment to match absolute voltages in the recorded signals. Gain stages in the commercial wireless hardware were designed to achieve the same theoretical total gain as the wired hardware. However, the precise cumulative gain in each recording system is sensitive to normal variability in device fabrication parameters. These resulted in a small but measurable and consistent gain difference between wired and wireless recorded signals. We calibrated absolute voltages by finding the gain multiplier that minimized differences in the NSS simulated spike

waveform amplitudes recorded in the wired and wireless bench tests. Based on these empirical measurements, we applied a +2.97% amplitude adjustment to the wireless data for comparisons in this study.

I. Digital Sample Integrity

Transmission data loss is a concern for all wireless systems. Here, wireless data loss occurred whenever the receiver did not find the expected sync word on any input channel. When this occurred, the current samples from all 96 electrodes were invalid and the previous valid frame was inserted into the data stream. This frame error was detectable in the recorded data as a back-to-back repeat of the previous 96-channel data sample. We quantified the number of frame errors in the received 20 kS/s wireless data by examining the 30 kS/s data recordings and accounting for the expected back-to-back sample repeats from the upsampling process. The practical effect of wireless data loss depends not just on the number of frame errors but on their distribution. Individual dropped frames (one 50 μs sample from each electrode) distributed sparsely throughout the data stream should be less detrimental to iBCI decoding than contiguous data loss [21]. We quantified the prevalence of longer intervals of data loss using the standard Severely Errored Seconds (SES) metric that reports the number of non-overlapping 1-second periods in which the proportion of data errors exceeds a predetermined threshold. Here, we applied a commonly used threshold of 50% to find all 1-second periods in which frame errors corrupted at least 500 ms of the data.

We interrogated the wired and wireless 30 kS/s recorded data for all instances of voltage changes exceeding a pre-determined biologically plausible slew rate (500 μV per 33 μs sample). These included bit-flips within single samples resulting in a large voltage excursion that returned to baseline in the following sample (or next sample after that in the case of upsample repeats), and large amplitude noise events extending over many samples before returning to baseline. We examined the degree to which each event was isolated to individual channels or observed simultaneously across many electrodes of the recording array.

III. Supplemental Videos

Audio playback in the supplementary videos has been removed in some cases to protect participants' privacy.

A. Supplementary Video 1: Participant T5 typing on-screen with the wireless iBCI.

This video (2 min 20 sec) shows participant T5 using the wireless iBCI to point and click in the Windows native on-screen keyboard to free-type in the Windows Notepad app. Typing performance is reported in the main text.

B. Supplementary Video 2: Participant T10 (tablet apps)

This video (2 min) shows participant T10 using the wireless iBCI to point and click in apps (Pandora, NCAA) on a tablet computer running Windows. The camera view shows T10 lying on his side in bed (with a red neck pillow). The tablet is mounted a meter or so away on a flexible holder. Note that the iBCI system is effective in the presence of ongoing music, talking, and the participant viewing the screen at a 90-degree rotation.

IV. Supplementary References

The bibliography below is a continuation from the main manuscript. Only references cited exclusively in this Supplementary Material are added here.

[69] J. Albites Sanabria, "BrainGate-enabled intracortical neural control of commercial tablet computers by individuals with tetraplegia," Thesis, Brown University, 2016.
[70] P. Nuyujukian *et al.*, "Performance sustaining intracortical neural prostheses," *J. Neural Eng.*, vol. 11, no. 6, p. 066003, 2014.
[71] F. Wood *et al.*, "On the variability of manual spike sorting," *IEEE Trans. Biomed. Eng.*, vol. 51, no. 6, pp. 912–8, 2004.
